# How Immediate and Urgent Needs Are Met

**Published:** 1919-08-02

**Authors:** 


### LEICESTER ROYAL INFIRMARY.

### How Immediate and Urgent Needs are Met.

The half-yearly meeting of the Leicester Royal Infirmary was noticeable not only from the record of work placed before the Governors, but for the difficulties with which the Board is grappling. The total admissions to the wards for the six months were 2,318, as compared with 2,116 in the corresponding period. In every category the numbers were higher. The expenditure was £23,034, and the income £18,718; the higher expenditure, £800 more than that of the corresponding period, is wholly attributable to the increased amount of salaries, wages, etc. The income showed a diminution of £3,000 due to the relinquishment of War Office grants now that the military wards have been vacated.

The New Orthopædic Department.

The need of a new Orthopædic Out-patient Department has been prominently before the governors for many months, and was pressed upon them by the visit of important representatives from the Pensions Ministry. Negotiations for the provision of a department with that Ministry proved abortive, so the Board determined to proceed at once with building and equipping the department. The Board's decision led to a most generous offer by the Freemasons of Leicestershire and Rutland to contribute £5,000 for the building of the department "In loving memory of those brethren who fell in the Great War," an offer which was accepted with readiness and gratitude. The building is being rapidly proceeded with, and the foundation-stone is shortly to be laid with Masonic Ceremonial. The pressing need of the department is proved by the increasing numbers attending the existing temporary department, amounting to 13,023 during the six months. The new department will be capable of dealing with 1,000 attendances per week, and the equipment will include rooms for Swedish remedial exercises, massage and electricity, whirlpool baths and other treatment.

A New Pathological Department.

The existing Pathological Department furnished 2,668 reports on various conditions and diseases during the six months. The new department, sorely needed during past years, is now being built, and in addition to up-to-date laboratories will include a new mortuary, *post-mortem* room, viewing chapel, refrigeration, etc. The pre-war cost of £5,000 will be doubled, but the department is so much needed that the builders have been instructed to proceed with all speed.

Venereal Diseases Department.

The attendances in this department on the figures of last quarter exceeds 13,000 attendances per year, and the treatment of this large number cannot be carried on in an existing department without great dislocation and inconvenience.

The medical staff have strongly recommended the Board to make provision in a separate department, which it is suggested should be built by the Local Government Board and the City and County Councils. Plans have accordingly been prepared and steps taken to urge consideration of the matter by the authorities.

Methods of Improvement.

The Board is looking to the future by setting out in the shape of "Peace Memorials" the means by which existing departments may be improved and new departments established. An isolation hospital, additional accommodation for the nurses, for the domestic servants, and the patients must be provided without delay.

Recognition is paid to the devotion of the staff, and particularly to the nurses, for their help in a time of unexampled difficulty. The institution, the report concludes, stood for that splendid medical service which has minimised the horrors of war and for the nurses who rose to the heights of their service. There was a great field open for generosity in acknowledging this medical and nursing service by undertaking the New Departments outlined above, in thankfulness for the conclusion of peace. The report was unanimously adopted. Mr. T. Fielding Johnson, Jr., was in the chair.

